# Protocol for the ORION trial (RadiO fRequency ablatION for haemorrhoids): a randomised controlled trial

**DOI:** 10.1007/s10151-022-02724-8

**Published:** 2022-11-09

**Authors:** C. Girling, M. J. Lee, D. Vimalchandran, D. J. Jayne, S. Stancliffe, A. Wailoo, M. Bradburn, D. Hind, M. Bursnall, L. K. Robinson, S. R. Brown

**Affiliations:** 1 Clinical Trials Research Unit, School of Health and Related Research, The University of Sheffield, Sheffield, UK; 2 Academic Directorate of General Surgery, Sheffield Teaching Hospitals NHS Foundation Trust, Sheffield, UK; 3 Countess of Chester Hospital NHS Foundation Trust, Chester, UK; 4 University of Leeds, Leeds, UK

**Keywords:** Haemorrhoids, Randomised controlled trial, Radiofrequency ablation, Surgical procedure

## Abstract

**Background:**

Haemorrhoids are common and can significantly impact the personal and working lives of individuals. Those with more severe symptoms and those not responding to conservative management may require surgery. Current surgical techniques are associated with a degree of postoperative discomfort which may delay return to normal activity. Recurrence is lower in more radical procedures but resulting pain is higher. Radiofrequency ablation (RFA) is a new technique that is gaining popularity and has several hypothesised benefits, including reduced pain and recurrence. However, available evidence is limited. A recent overview from the National Institute for Health and Clinical Excellence recommended more research, in the form of randomised controlled trials, be carried out before further investment is made by national health services. Our aim is to assess whether RFA is at least as good in terms of recurrence as existing surgical interventions, but superior in terms of pain, for patients with symptomatic grade II and III haemorrhoids.

**Methods:**

The RadiO fRequency ablatION for haemorrhoids (ORION) trial will be a pragmatic multicentre patient/assessor-blind parallel group-controlled trial with economic evaluation. The target sample size is 376 participants (188 per arm) and is based on two co-primary endpoints: (i) a non-inferiority design for recurrence and (ii) superiority design for pain at seven days. Participants with grade II or III haemorrhoids will be recruited in 16 National Health Service hospitals and randomised (1:1) to either RFA or surgeon’s choice of surgery.

**Conclusions:**

Results will inform future practice for the treatment of grade II–III haemorrhoids and provide evidence for national health services on future investments in RFA.

**Trial registration:**

ISRCTN14474552.

## Introduction

Haemorrhoids result from pathological changes to the vascular tissue that forms the anal canal and cause symptoms including discomfort and bleeding, frequently resulting in patients presenting for review in surgical clinics. As many as 1 in 3 individuals are affected [1], with over 20,000 operations carried out each year in England alone [2]. Haemorrhoids can cause significant disruption to the personal and working lives of the affected population, as treatment often involves frequent hospital visits and extended recovery periods.

Treatment is determined by the extent of the symptoms and the degree of prolapse, and expert opinion and current guidelines both promote a tailored approach to treatment selection [3]. A number of surgical interventions are currently available to patients, including haemorrhoidal artery ligation (HAL), stapled haemorrhoidopexy, and techniques for surgical excision, all of which are routinely performed under regional or general anaesthetic [3]. Post-surgery discomfort is associated with all treatment options. In the longer term, recurrence of symptoms is not uncommon. Most recent evidence suggests that open haemorrhoidectomy is the most painful in the short term, but it is the least expensive, and better quality of life is observed in the long-term [4–6]. At present, all three interventions are recommended for use [3].

An alternative treatment might be radiofrequency ablation (RFA), which has emerged as an option for patients with haemorrhoids [7]. Like the surgical interventions discussed, RFA is generally suitable for those where office procedures such as Rubber Band Ligation (RBL) have been unsuccessful, or for those with more significant prolapse which is unlikely to respond to less radical intervention [7]. Compared to excisional treatments, RFA has been proposed as a faster procedure with more rapid recovery as it does not excise tissue or generate excess heat. Together, these features of RFA have led to its increasing popularity. However, existing evidence is limited to small cohort studies in specialist settings [8, 9], and pertinently, the promising longer term effects have not been rigorously tested in a randomised comparison.

Evidence on RFA was summarised in a National Institute for Health and Care Excellence (NICE) overview [7]. This concluded that the quantity and quality of existing evidence on the safety and efficacy of RFA for haemorrhoids is inadequate and that further randomized controlled trials (RCTs) are encouraged with outcomes including pain, secondary haemorrhage, recurrence rate, the need for repeat procedures and quality of life measurements.

If more rigorous evidence can demonstrate that RFA can achieve outcomes at least as effective as current recommended interventions, whilst showing superior outcomes for the patient in terms of reduced inconvenience and more rapid recovery, then RFA can be legitimately incorporated in the treatment algorithm for haemorrhoids, particularly if more cost-effective. However, if the RFA procedure does not meet these criteria, health services will be able to disinvest.

The primary aim of this trial is to assess whether radiofrequency ablation is at least as good as existing methods for treating haemorrhoids in terms of recurrence but superior in terms of postoperative pain.

## Materials and methods

This protocol has been prepared with reference to the Standard Protocol Items: Recommendations for Interventional Trials (SPIRIT) guidelines. The trial will be a pragmatic multicentre patient/assessor blind parallel group individual participant randomised (1:1 allocation) controlled trial with economic evaluation. The trial will take place in clinical sites in the UK which offer elective surgery for haemorrhoids.

### Eligibility criteria

Participants will be adults aged 18 years or over with symptomatic second- or third-degree haemorrhoids [10]. Potential participants will have failed conservative managements (diet and lifestyle changes) and requested further intervention, or have either failed one rubber band ligation (RBL) procedure, have haemorrhoids considered inappropriate for RBL treatment, or their surgeon considers the haemorrhoids suitable for surgical intervention.

Patients with certain pre-existing medical conditions will be excluded, including known perianal sepsis, inflammatory bowel disease, anal or colorectal malignancy, pre-existing sphincter injury. In addition, patients with an immunodeficiency (human immunodeficiency virus or other medical cause), unable to have general or spinal anaesthetic, or currently taking warfarin, direct oral anticoagulants that cannot be safely stopped prior to surgery or with any other hypocoagulability condition that may increase the risk of bleeding, or have a pacemaker, will be excluded. Pregnant women and patients who are unable to give full informed consent (due to mental capacity barriers) will also be excluded.

### Intervention

The intervention is RFA (using the Rafaelo^®^ device, Modern Aesthetic Solutions Ltd, UK). The control arm is surgeon choice of surgery, which could be one of stapled haemorrhoidopexy, HAL or haemorrhoidectomy (consistent with international guidelines [3]). ORION will be a pragmatic trial; all procedures will be conducted as per standard care at individual hospitals. RFA has NICE approval where there are arrangements for clinical governance, consent, and audit or research [7].

### Patients randomised to RFA

RFA is available to the UK National Health Service (NHS) through the Rafaelo^®^ device. A special needle probe is inserted into the haemorrhoidal cushion, through which radiofrequency energy is applied, aiming to restrict its blood supply causing it to necrose autoamputate, relieving the patient of their symptoms. In the UK, RFA is generally performed under general anaesthesia with the patient positioned in lithotomy. It can be performed under local anaesthetic. A proctoscope (F care systems, Antwerpen, Belgium) with a simple vent on one side, through which a single haemorrhoidal tissue protrudes, is placed in the anal canal. At a level approximately 5 mm above the dentate line, the submucosa of haemorrhoidal tissue is infiltrated with approximately 1 ml of bupivacaine 0.25%. In addition to achieving local anaesthesia, this step creates a fluid barrier to prevent the transmission of heat to the internal anal sphincter muscle. The Rafaelo^®^ device and associated HPR45i probe (F care systems, Antwerpen, Belgium) are used to deploy RFA energy of 4 MHz frequency to the haemorrhoidal tissue. The tip of the probe is inserted fully into the haemorrhoid tissue approximately to a depth of 5–10 mm, at an approximately 30° angle to the tissue surface. The haemorrhoidal tissue is tilted away from the submucosal layer. The application of RFA is continued until the tissue exhibits whitish discolouration, after which the energy is applied to the external surface of the haemorrhoidal tissue to optimize tissue desiccation. An optimum of 3000 J with a power setting of 25 W is applied to an individual haemorrhoidal tissue at one time. A cold saline-soaked tonsillar swab is immediately applied to the surface of the haemorrhoidal tissue. Any bleeding is controlled by inducing coagulation using the radiofrequency probe [8].

All surgeons involved in the study will have completed training and will have experience of at least five procedures prior to recruiting to the study.

Participants will receive standard supportive care for a surgical intervention, as per local procedures. This will usually be in the form of available clinical contact for any concerns as well as access to clinicians responsible for the participant care if appropriate.

### Patients randomised to surgeon’s choice of other procedures currently available in the UK

The alternative therapies vary, and often depends on the preference of the surgeon. Stapled haemorrhoidopexy, HAL and haemorrhoidectomy are all available in the UK NHS and appropriate conventional therapies according to international guidelines [3]. The control arm will therefore comprise the ‘surgeon’s choice’ of operation based on one of these three options.

### Outcomes

The co-primary outcome measures are recurrence at 12-months post-procedure, defined as per the HubBLe trial [6], and average Numeric Pain Rating Scale (NPRS) at 7 days post-procedure [11].

Secondary outcome measures include:

1. NPRS (1, and 21 days 6 weeks and 1 year post-procedure)
2. Number of days of work lost (measured by research nurse at 6 weeks post-procedure)
3. Persistence of haemorrhoidal symptoms at 6 weeks post-procedure
4. Haemorrhoid severity score [12]
5. EuroQol 5 Dimension 5 Level (EQ-5D-5L)[13] (days 1, 7 and 21, 6 weeks, 1 year post-procedure)
6. Self-report, 7-item Vaizey incontinence score (6 weeks, 1 year post-procedure) [14]
7. Health and social care resource use questionnaire (6 weeks, 1 year post-procedure)
8. Complications (see Table 1)
9. Cost

**Table 1:**
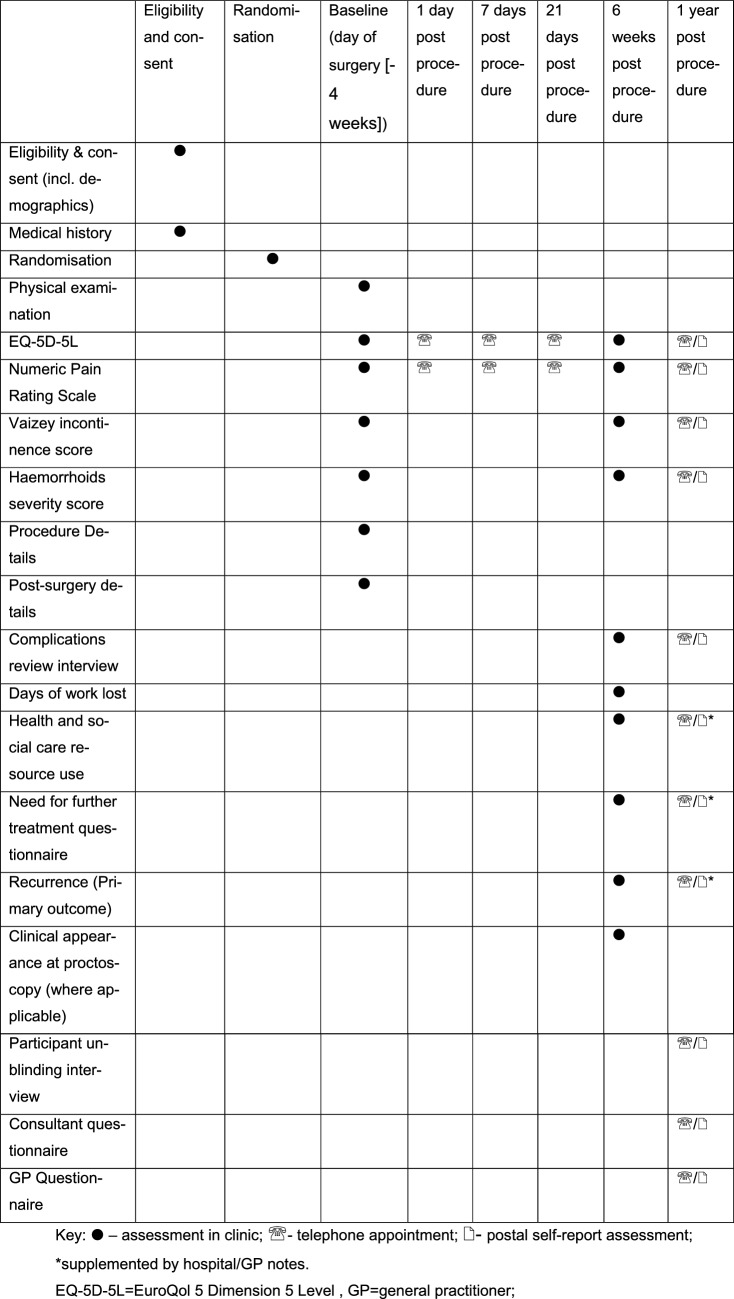
Use of assessment instruments during the trial

Outcome data (Table 1) will be collected by research nurses, consultants and specialist nurses, either in-person at Standard Operating Procedures (SOPC) (baseline data, 6 weeks follow-up if the usual clinic is face-to-face) or by telephone questionnaires (day 1, 7, 21 and 12 months, and at 6-weeks if the usual clinic format is by telephone) or by completed postal questionnaire (12 months postoperatively). In addition, the 12-month data on recurrence, complications, resource use and need for further treatment will be supplemented by hospital/GP note review. In the instance of disparity of responses between the consultant/hospital notes, serious adverse events (SAE) reports, consultant and general practitioner (GP) records and the participant, the chief investigator will act as the ultimate arbitrator. In the scenario of no response from the patient with regard to recurrence but with another procedure to treat haemorrhoids in their medical notes, this will be recorded as a recurrence. Measuring recurrence based on SAEs will be treated on a case-by-case basis. For example, bleeding within 2–3 days of the procedure which subsided would not be categorized as a recurrence, but uncontrolled episodes later would.

Participant study data will be recorded on study-specific case report forms (CRFs) and patient questionnaires and then entered onto a remote web-based data capture system, transferring data to Sheffield Clinical Trials Research Unit (CTRU) for analysis. All aspects of data management will be provided by the Sheffield CTRU in accordance with their own standard operating procedures.

### Participant recruitment and timeline

Patients will be recruited from 16 UK NHS hospitals over a 14-month recruitment period. The patients care team will conduct an initial case note review for eligibility. Potential participants will receive an approved Participant Information Sheet and given the opportunity to ask questions from both the surgical and research team at their hospital before enrollment into the trial. Participants will be approached either by the local Principal Investigator (PI) or a delegated team member with the appropriate Good Clinical Practice (GCP) training. Participants will give written consent either by remote postal consent, or face to face at surgical outpatient’s clinics. Reasons for non-consent will be recorded where possible and monitored by the study team.

After consent participants will be individually randomised in equal proportions at all centres using a remote, web-based randomisation system.

- *Group A*: RFA using the Rafaelo^®^ device or
- *Group B*: Surgeons’ choice of other procedures currently available in the UK NHS.

**Fig. 1:**
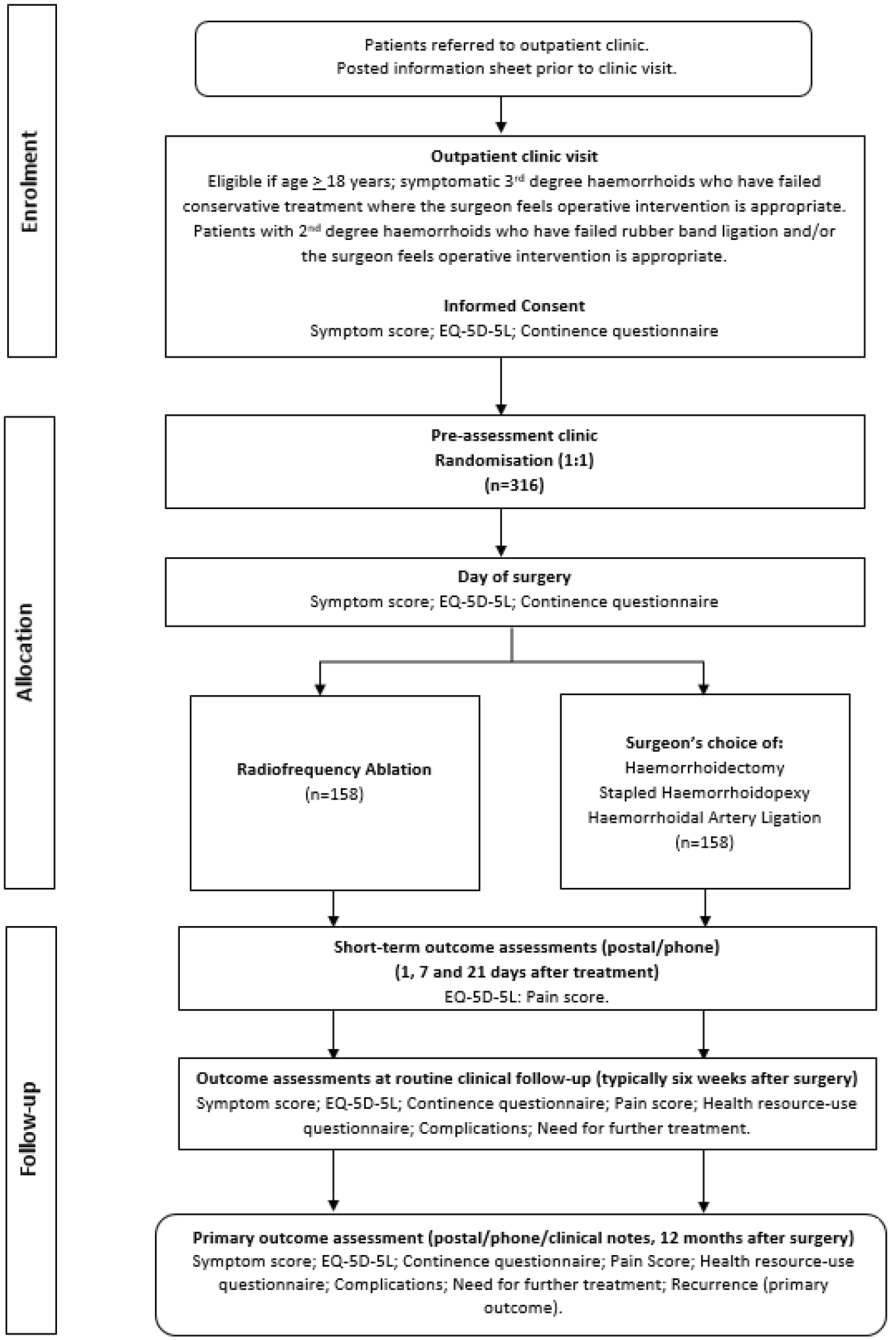
Participant study flow diagram for visits and data collection. *EQ-5D-5L* EuroQol 5 Dimension 5 Level

Baseline data will be collected on the day of surgery or up to 4 weeks before surgery. Details of the procedure will be recorded during or shortly after the surgery. Following the procedure data will be collected to establish scores of pain, as well as which patients have further treatment for recurrent symptoms or complications. This will be achieved using follow-up telephone questionnaires at 1, 7 and 21 days; at a clinic visit around 6 weeks after the intervention; and by interrogating hospital records, asking the patients consultant and writing to the patients GP at the 12-month follow-up. A summary of the participant involvement can be found in Fig. 1.

A patient can withdraw from the trial at any point without giving reasons. Data collected up to the point of withdrawal will be retained. The date of withdrawal will be recorded on the case report form and the web-based data capture system. A summary of data collection can be found in Table 1.

### Sample size

The target sample size is 376 participants (188 per arm) and is based on two co-primary endpoints: (i) a non-inferiority design for recurrence and (ii) superiority design for pain at 7 days. Previous research has demonstrated RFA is associated with a recurrence rate of 4% to 15%, compared with 15% for haemorrhoidectomy and 25–30% for HAL. Our patient panel members have advised us that RFA would be acceptable if we could rule out a 10% increase in recurrence, which we have used as our non-inferiority limit, accompanied by a reduction in pain. The trial will recruit 376 participants (188 per arm), which provides 90% power to declare non-inferiority based on a 15% drop out, an Intraclass Correlation Coefficient (ICC) of 1% among 16 surgeons, a one-year recurrence rate of 15% for intervention and 20% for usual care, a non-inferiority limit of 10% and a one-sided 2.5% significance level. These assumptions are heavily based on our previous HubBLe trial which found a 12% drop-out in the HAL surgery arm and a zero ICC for 12-month recurrence [6]. A sample size of 376 ensures a 90% power to detect a minimal clinical importance difference (MCID) of 0.6 points (1/3rd of a standard deviation) in Numeric Pain Rating Scale-reported pain at 7 days at the two-sided 5% level assuming 5% missing data, a correlation of 0.5 between baseline and follow up and an ICC of 1%. No adjustment for multiple testing is necessary since RFA will need to demonstrate significance on both endpoints.

### Assignment of intervention

A web-based randomisation system will generate random assignment with stratification by hospital. Research staff at hospitals will enrol participants on the randomisation system. Outcome assessors, statistician and participants will be blinded to allocation. In the event a patient or clinician needs to reveal allocation this is accessible through the unblinding procedure on the randomisation system.

### Statistical and health economic analysis

Analyses of recurrence will be performed using generalised estimating equations (GEE) using the binomial family and the logit link treatment arm and grade of haemorrhoid as fixed effects, with surgeon being incorporated as a clustering term. The difference in proportions and its associated CI will be derived using the delta method [15]. Pain scores at day seven will be analysed using GEE with an identity link; the fixed effect covariates will be treatment arm, grade of haemorrhoid and pre-procedure pain rating, with surgeon again incorporated as a clustering term. Secondary endpoints will be analysed analogously. Unadjusted analysis (difference between arm and 95% CIs) will be reported alongside adjusted analysis.

Safety will be assessed by (i) post-surgical complications and (ii) postsurgical complications leading to SAE, both of which will be summarised for each arm by the number of participants experiencing (a) each complication type at least once and (b) any complication at least once.

The primary analysis will use the Intention to Treat (mITT) population (we do not follow-up participants who withdraw before surgery). For the primary outcomes only, Per Protocol (PP) and As Treated (AT) populations will be considered for sensitivity analysis. There is no a priori defined sensitivity analysis for secondary outcomes.

We will use an interaction statistical test between intervention arm and subgroups to directly examine the strength of evidence for the between arm difference varying between subgroups for the primary outcomes. Age and grade of haemorrhoid will be the only a priori defined sub-groups to be considered for interaction test. Sub-group analysis will be performed regardless of the statistical significance on the overall intervention effect.

Case and item missing data will be examined and multiple imputation methods will be used to reduce bias due to any missing responses in the analyses. Where appropriate, modelling methods that generate robust standard errors (SEs) in the presence of missing data will be considered.

We will separately calculate the primary outcomes for each of the three surgical options in the control arm and will calculate for each of them the difference (and associated 95% CI) between (1) RFA and haemorrhoidopexy; (2) RFA and, HAL; and (3) RFA and haemorrhoidectomy. It should be noted that these are exploratory (and non-randomised) comparisons and not subject to the benefits of randomisation; as the characteristics of the control surgery sub-groups may not be balanced when compared to RFA.

For the cost-effectiveness analysis, the area under the curve method will be used to analyse EQ5D and estimate Quality Adjusted Life Years (QALYs) for each individual.

Resource use is collected for the following categories: the direct costs of surgery, the costs of treating recurrence, other complications, and any other relevant NHS resource use. An NHS perspective will be used for costing resource use.

Unit costs, to apply to each category of resource use will come from sources widely used in economic evaluations such as NHS Reference Costs, NHS supply systems, British National Formulary and ‘Unit Costs of Health and Social Care’ published by the PSSRU. Where necessary, we will supplement these unit costs with local sources such as the finance departments of participating hospital trusts.

The analysis will use multiple imputation methods for missing data. For each individual we will estimate total costs and QALYs over the 12-month follow-up period. The mean costs and QALYs for each comparator will be estimated and regression analysis used to adjust for baseline characteristics of patients assigned to each arm. Bootstrap methods will be used to generate the cost-effectiveness plane and associated cost-effectiveness acceptability curve.

## Monitoring

### Safety monitoring

Any complications that occur following the intervention will be identified on the ‘Procedure details’ CRF, at the 6-week clinical visit, and at the 12-month follow-up. If there are any clinical concerns (including mental distress) about a participant, these will be referred to the appropriate clinical team for further investigation. SAEs will be reported in accordance with local SOPs, which comply with National Research Ethics Service & GCP [16].

### Auditing

Data monitoring will be undertaken periodically by the steering committee and management groups to identify missing data and potential outlaying/erroneous data. Data issues will be identified and actioned by the management group.

### Ethical approval

The research has been approved by London-Queen Square NHS Research Ethics Committee (ref 21/LO/0762). Amendments to the protocol will be submitted to the Health Research Authority and Research Ethics committees as required and circulated to relevant parties.

### Confidentiality

All data will be handled in according to General Data Protection Regulation (GDPR) 2018 principles [17]. Data will be held securely and will be accessible only by members of the research team.

### Patient and public involvement

People with lived experience of haemorrhoids were involved in the design and the development of the ORION trial. Continued patient and participant representation was incorporated through the Trial Management Group and Trial Steering Committee.

### Dissemination

The ORION trial group, comprising of all individuals who contribute to the trial (see supplementary publication and dissemination plan for details) will disseminate the findings through peer-reviewed journals, and the Association of Coloproctology of Great Britain and Ireland.

## Discussion

Haemorrhoid disease is common worldwide and represents a significant burden on patients and public health services. This combined with the desire for an effective treatment that is tolerable, safe and convenient for the patient has led to numerous innovations over the last three decades. Many such innovations are adopted into practice with overinflated claims about efficacy and tolerability and subsequently found to be lacking. Developmental costs for these innovations are passed onto stretched public health services and often have significant economic implications. It is essential that such innovations are encouraged but that the efficacy and tolerability of each are tested thoroughly before general adoption.

RFA is at risk of being such an innovation. It appears to have several theoretical advantages. A low intensity procedure that may be carried out under local anaesthetic; low heat coagulation and less damage to surrounding structures resulting in theoretically less pain; and some cohort data suggesting efficacy [7]. However, this data is subject to significant bias [7]. Cost is significant and there is no cost-efficacy data to justify adoption into public health services [7]. Nevertheless, the procedure is gaining interest in the UK [7], therefore these data are urgently required.

Our proposed trial meets all the requisites suggested by NICE [7]. It will be the largest randomised clinical trial to assess whether RFA is as good as other current surgical techniques for treating grade II and III haemorrhoids, in terms of recurrence and pain. A rigorous governance structure incorporated in the study design will ensure robust results with minimal potential for bias. Previous experience with both the Hubble (ISRCTN41394716) and Ethos (ISRCTN80061723) trial means that we are confident of both completing the trial and delivering meaningful data that will inform the surgical community [5, 6].

If the trial does demonstrate positive results in terms of efficacy and tolerability, then it may be legitimately incorporated into the armamentarium of treatments for haemorrhoids, subject to cost effectiveness.
